# Supplementary figures and images for: Improving Rice Quality by Regulating the Heading Dates of Rice Varieties without Yield Penalties
Source: Plants (Basel). 2024 Aug 10;13(16):2221. doi: 10.3390/plants13162221 (PMC11360702; doi:10.3390/plants13162221)

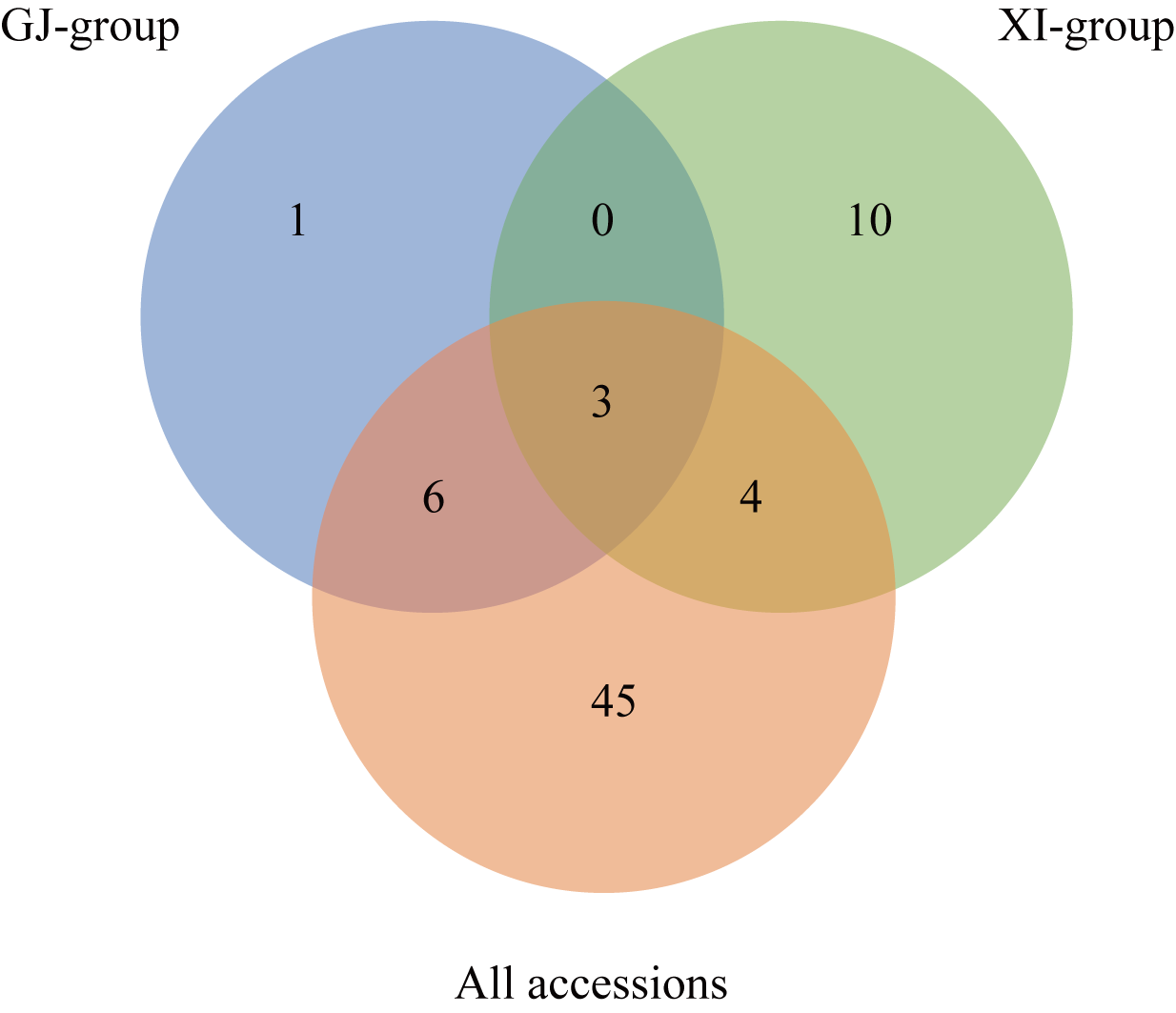

Supplement: Supplementary file 1 [file plants-13-02221-s001.zip › Figure S1.tif]

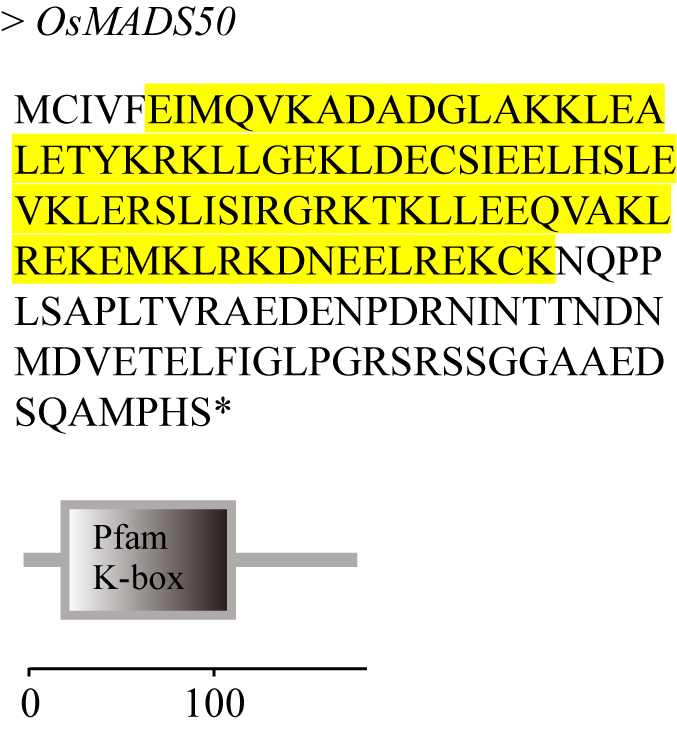

Supplement: Supplementary file 1 [file plants-13-02221-s001.zip › Figure S2.tif]

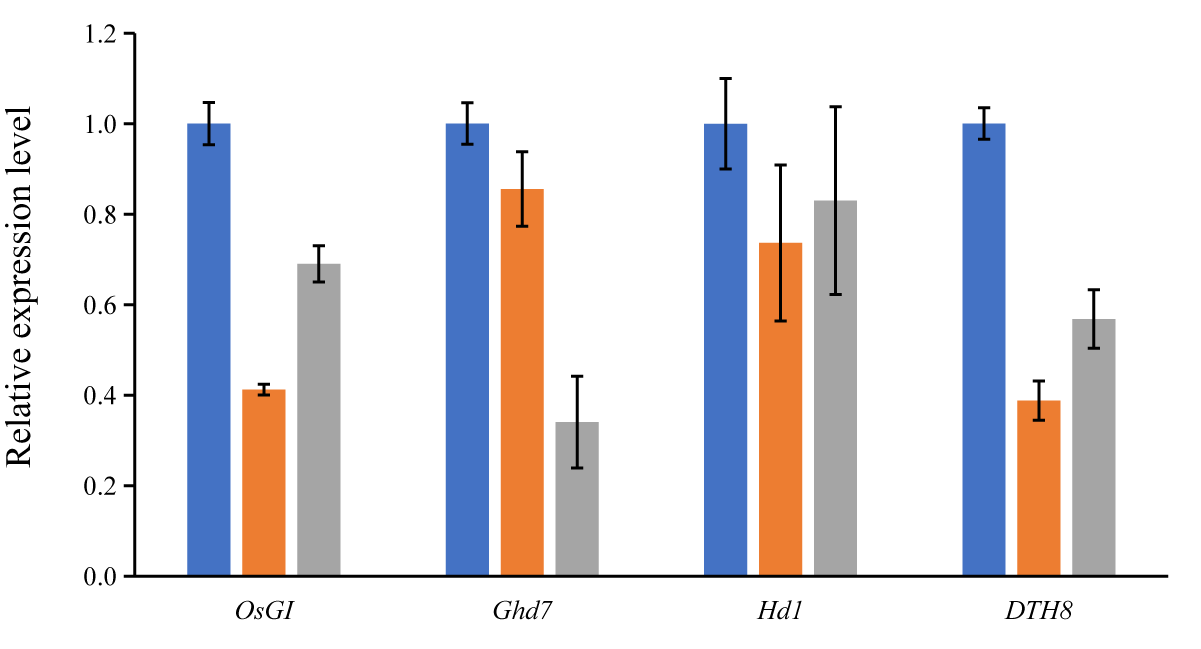

Supplement: Supplementary file 1 [file plants-13-02221-s001.zip › Figure S3.tif]

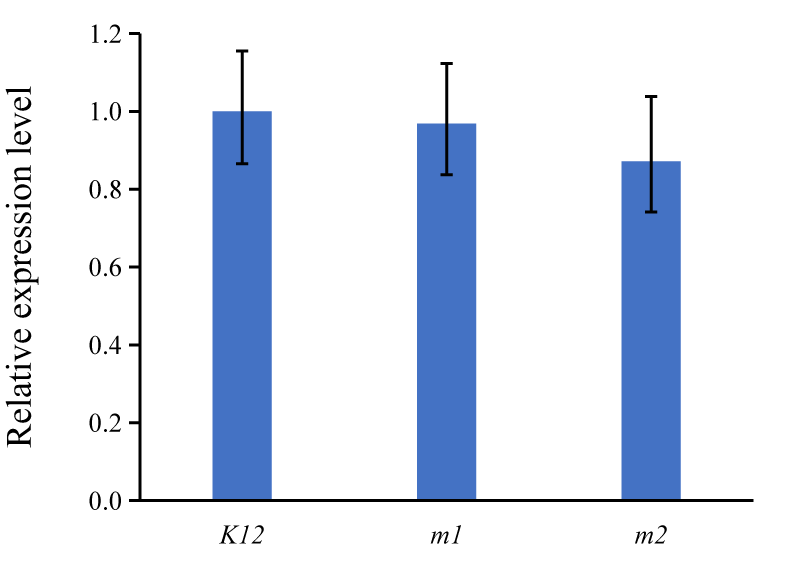

Supplement: Supplementary file 1 [file plants-13-02221-s001.zip › Figure S4.tif]

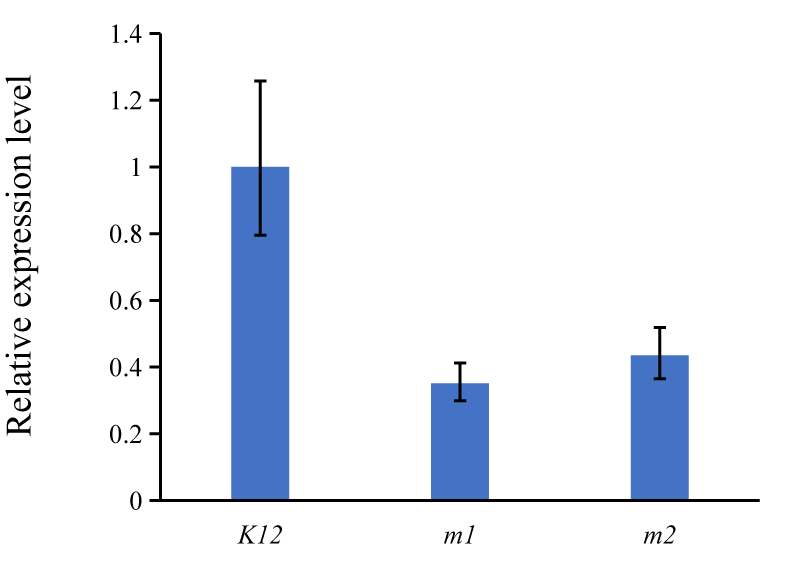

Supplement: Supplementary file 1 [file plants-13-02221-s001.zip › Figure S5.tif]
